# The Effect of a Fat-Restricted Diet in Four Patients with Familial Chylomicronemia Syndrome: A Long-Term Follow-Up Study

**DOI:** 10.3390/children8111078

**Published:** 2021-11-22

**Authors:** Alexandra Thajer, Gabriele Skacel, Charlotte de Gier, Susanne Greber-Platzer

**Affiliations:** Department of Pediatrics and Adolescent Medicine, Division of Pediatric Pulmonology, Allergology and Endocrinology, Medical University of Vienna, 1090 Vienna, Austria; gabriele.skacel@akhwien.at (G.S.); charlotte.degier@meduniwien.ac.at (C.d.G.); susanne.greber-platzer@meduniwien.ac.at (S.G.-P.)

**Keywords:** familial chylomicronemia syndrome, FCS traffic light table, dietary management, lipoprotein lipase deficiency, primary chylomicronemia, triglycerides, pediatric

## Abstract

(1) Background: Familial chylomicronemia syndrome (FCS) is a very rare autosomal recessive disorder characterized by severely elevated triglycerides and clinical symptoms in early childhood mainly presenting with abdominal pain, acute pancreatitis and hepatosplenomegaly. Primary treatment is a lifelong very strict low-fat diet, which might be challenging in pediatric patients. So far, data about children with FCS are rare. The aim of this study was to show the familial chylomicronemia syndrome traffic light table for pediatric patients and to assess the dietary fat intake and impact on triglycerides in children with FCS. (2) Methods: We performed a retrospective analysis in four children (50% male) affected by FCS from the Department of Pediatrics and Adolescent Medicine, Medical University of Vienna between January 2002 and September 2020. (3) Results: The four patients presented with classical FCS symptoms and showed baseline triglycerides (TG) exceeding 30,000 mg/dL in two patients, 10,000 mg/dL and 2400 mg/dL in one patient each. After diagnosis, fat percentage of total daily caloric intake was decreased and resulted immediately in triglyceride reduction. In all patients, FCS was genetically confirmed by mutations in genes encoding lipoprotein lipase. Acute pancreatitis and hepatosplenomegaly disappeared under the fat-restricted diet. A FCS traffic light table was developed as a dietary tool for affected families. (4) Conclusions: A restriction of dietary fat between 10% to 26% of the total daily caloric intake was feasible and effective in the long-term treatment of genetically confirmed FCS in children and could reduce the risk for acute pancreatitis. The dietary tool, the pediatric FCS traffic light table and the age-appropriate portion sizes for patients between 1 to 18 years, supports children and their parents to achieve and adhere to the lifelong strict low-fat diet.

## 1. Introduction

Familial chylomicronemia syndrome (FCS) is a rare autosomal recessive disorder of the lipoprotein metabolism with a prevalence of one to two in a million in the population [1,2]. FCS usually manifests in early childhood and is characterized by the presence of large triglyceride-rich lipoprotein particles called chylomicrons in the plasma that mainly originate from dietary fat [3]. Diagnosis is based on triglyceride levels > 1000 mg/dL (>11.3 mmol/L) presenting as lipemic plasma and clinical symptoms with recurrent abdominal pain and acute pancreatitis [1,3]. Other clinical signs are eruptive xanthomas, especially in the knees, buttocks and arms, lipemia retinalis, hepatosplenomegaly, failure to thrive, vomiting and nausea [3]. The diagnosis of familial chylomicronemia syndrome is confirmed by genetic mutations of the lipoprotein lipase (LPL) and other genes functioning as cofactors for LPL [3].

Fibrates, niacin, statins and omega-3 fatty acids are possible medications to reduce triglycerides in general, but because of the absence of active lipoprotein lipase or necessary cofactors, usually have no effect in FCS [3,4]. Volanesorsen, an antisense-mediated inhibitor of apolipoprotein CIII mRNA, might be effective in the treatment of FCS, but is not approved in children and adolescents so far [5]. Thus, the only therapeutic option for pediatric FCS patients comprises a strict fat-reduced diet [6,7]. Current guidelines have recommended a limitation of fat intake lower than 10–15% of total daily energy intake throughout life span [6]. So far, there are no data available on whether these guidelines can actually be implemented for pediatric FCS patients and, above all, whether they can be adhered to for a lifetime.

Triglyceride levels more than 2000 mg/dL (>22.6 mmol/L) are associated with an increased risk of acute pancreatitis, which occurs in 15–20% in hypertriglyceridemia patients [8,9]. Up to 25% of infants with FCS can suffer from acute pancreatitis episodes [10]. Therefore, early diagnosis and therapy are most important to prevent acute pancreatitis, which means a lifelong strict fat-reduced diet to reduce plasma triglycerides. The intention of a nutritional therapy is to decrease triglycerides <2000 mg/dL. Furthermore, the achievement of triglyceride levels of <1000 mg/dL with a diet is a great success [3,6]. Data on children with FCS are rare and mainly based on individual case reports [11,12,13,14].

The implementation of a fat-restricted diet in pediatric patients is challenging. Moreover, nutritional requirements vary considerably from infants to adolescents and particular individual needs must be taken into account. A dietary tool is missing that lists and categorizes food and beverages for children, adequate for age groups between 1 to 18 years, to support families to create a suitable diet for children with FCS to improve clinical manifestations and further to reduce and prevent pancreas atrophy, and hepatosplenomegaly. Currently, there are no studies that show long-term data on dietary intake and the impact on triglycerides in a young FCS population.

The aim of the study was to present the familial chylomicronemia syndrome traffic light table for pediatric patients and to assess the dietary fat intake and impact on triglycerides in children with familial chylomicronemia syndrome. Additionally, to report on changes in development, growth, clinical FCS manifestations and nutrition.

## 2. Materials and Methods

### 2.1. Study Design

The study was a retrospective data analysis from four children with FCS aged <18 years, who were in regular medical control at the outpatient clinic of obesity, lipometabolic disorders and nutritional medicine from the Department of Pediatrics and Adolescent Medicine, Medical University of Vienna between January 2002 and September 2020.

Data collection comprised baseline values from the first visit at the outpatient clinic (visit 1), after implementation of a fat-restricted diet (visit 2), and follow-up visits for several years (visit 3, 4, 5 and 6). The visits with documented blood parameters as well as the 3-day food records, which were fully completed and analyzed, were used. The local ethical review board approved this study (MUV, EC Nr: 1331/2016).

### 2.2. Clinical Anamnesis, Medical History and Blood Measurements

Family history, clinical diagnosis and physical examination were routinely recorded at the outpatient clinic. At each visit, body weight (kg) and body height (cm) were measured and the Austrian body mass index (BMI) (kg/m²) percentiles were used [15,16].

Blood sampling was performed at each visit and triglycerides and total cholesterol concentrations were measured after an overnight fast. In all patients, abdominal ultrasonography (liver, pancreas, gall bladder and splenic) was performed and genetically testing was conducted for sequencing of genes associated with dyslipidemia.

### 2.3. Diet

For each patient our dietitian developed an individualized diet plan. The focus was primarily to decrease the fat intake considering adequate intake of energy, essential fatty acids, fat-soluble vitamins and micronutrients. When age-appropriated weight gain was not achieved and to guarantee adherence to the diet, medium-chain triglycerides (MCT) and maltodextrin were substituted. In infants, special infant formula with very low-fat content was used. The infants received either Milupa basic-F (Nutricia, Austria), which contains 0.09 g fat per 100 mL (<0.1% fat, and not any MCT) or Monogen (Nutricia, Austria), which comprises 2.2 g fat per 100 mL (a low-long chain triglycerides (LCT; 16%) amount and high-MCT (84%) ratio).

The introduction of complementary foods, defined as solids and liquids other than breast milk or infant formula, and the further choice of food was based on the FCS traffic light table (Table 1).

This FCS traffic light table was specially developed and used for the training of parents and their children with familial chylomicronemia syndrome at the outpatient clinic of obesity, lipometabolic disorder and nutritional medicine, Department of Pediatrics and Adolescent Medicine of the Medical University of Vienna. The Optimized Mixed Diet (OMD) of nutrition for healthy children and adolescents with age-adapted portion sizes was used as basis for this traffic light table [17,18,19]. OMD represents in a pyramid multiple food groups in three categories: green (ample/liberal), yellow (moderate) and red (sparing/occasional) [17]. This OMD-concept was modified for children and adolescents with familial chylomicronemia syndrome as well as to the individual tolerance of each patient. For individual tolerance, several aspects must be considered such as availability of dietary products, feasibility of dietary recommendations, and which triglyceride values can actually be achieved with the diet. The individual tolerance represents the maximum dose of fat intake, whereby the triglycerides are still within an optimized range of about 1000 mg/dL to prevent any side effects like pancreatitis. Parents and patients, if possible and interested, were regularly trained in food selection and meal composition, but also in fat-reduced cooking or cooking without cooking oil (e.g., steam cooking and using an air fryer). Parents and children learned to use the food FCS traffic light table to categorize nutrition into three groups. Green-marked food and drinks are recommended and can be eaten without restriction, yellow-marked food should be consumed according to the age-appropriate portion sizes and red-marked food should be avoided. The “green traffic light area” contains almost fat-free food, like bread without nuts and seeds, potatoes, rice and other grain, vegetables or fruits. The “yellow traffic light area” contains low-fat food, such as low-fat vegetarian spreads, low-fat yoghurt, low-fat sausages or low-fat meat and fish. The yellow-marked food is based on age-appropriate quantities. The age-adapted portion sizes of the food groups are summarized in Table 2. The household size as well as the child’s hand to determine 1 portion size support the understanding of quantities and improves the implementation of the diet.

The “red traffic light area” contains fat-rich food consisting of long-chain fatty acids, like cooking oils, high-fat meat, high-fat sausages and high-fat sweets such as chocolate. Red-marked food, even in small amounts, could have a significant effect in individuals with FCS on the plasma triglyceride levels with extreme increase and therefore should be avoided. For more visualization educational material is available for patients and families as colored tables containing actual pictures of very low-fat food products offered in the local supermarkets.

The dietitian trained parents and patients to protocol and complete 3-day food records. At control visits, food records were analyzed together with the dietitian for possible food adaptions. The adherence to the prescribed diet plan was evaluated by the 3-day food records analyzed with the nutritional software nut.s (dato Denkwerkzeuge, Austria, v1.32.77) in combination with the current triglyceride level.

### 2.4. Statistical Analysis

Each patient is shown individually in order to see the course and the changes of the blood parameters and nutrient intake. All data obtained of measurements at each visit are presented in the tables (Table 3, Table 4, Table 5 and Table 6). Results are expressed as numbers, absolute frequencies and proportions. Values for triglycerides, dietary fat intake and FCS symptoms were compared by the Spearman correlation. *p*-values of <0.05 were considered statistically significant. Statistical analysis was performed using the software Statistical Package for Social Science SPSS (SPSS Inc., Chicago, IL, USA, version 24.0).

## 3. Results

### 3.1. Patients

**Figure 1:**
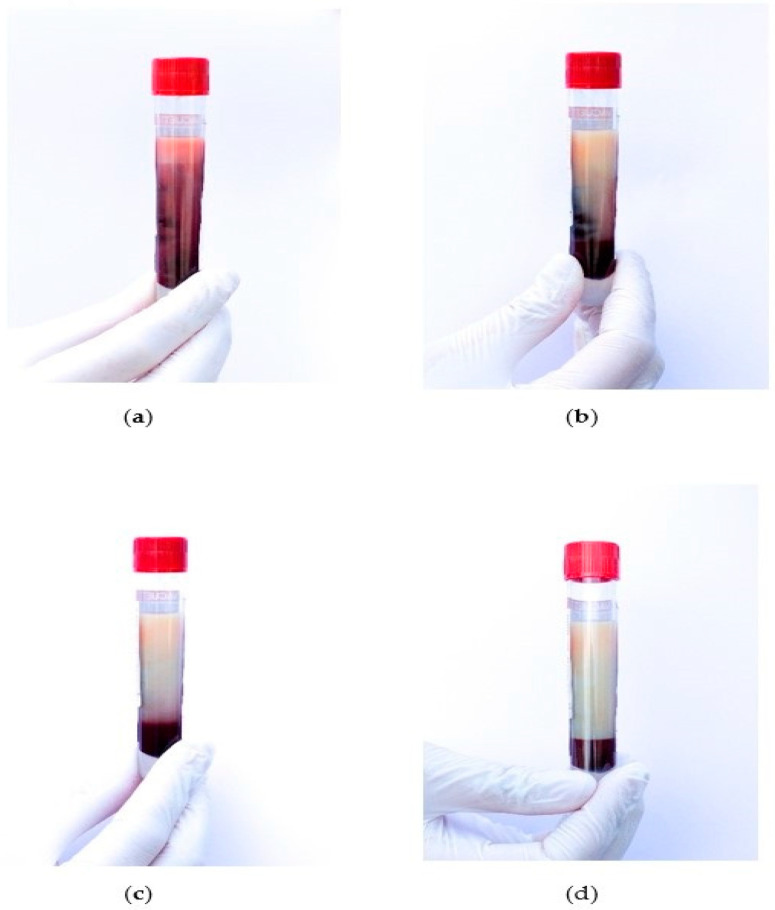
Lipemic plasma distribution in an EDTA tube (**a**) immediately after blood sampling (**b**) after 1 h (**c**) after 2 h (**d**) after 3 h.

In total, four individuals (*n* = 4, 50% male) were included in the study. Each patient is presented in a separate table (Table 3, Table 4, Table 5 and Table 6). At baseline, all patients showed clinical symptoms of familial chylomicronemia syndrome and plasma appeared lipemic due to increased triglyceride and chylomicron levels (Figure 1a–d).

Early intervention can prevent further complications and the greatest impact could be achieved immediately after diagnosis and under a very strict dietary regime in all patients. The Spearman correlation showed a significant positive correlation between triglycerides and dietary fat intake in patient 1 (r_s_ = 0.943; *p* < 0.01). In two patients, patient 2 and 3, a strong negative correlation between triglyceride levels and FCS symptoms was noticed (r_s_ = 0.828; *p* < 0.05). All patients showed fluctuations in their fat intake, which directly affected their triglyceride levels (Table 3, Table 4, Table 5 and Table 6).

### 3.2. Genetics

Genetic analysis was confirmed in four patients with FCS mutations in the lipoprotein lipase gene. Three patients carried different homozygous mutations on the LPL gene, these were c.337t > C (*p*.Trp113Arg) and c.557G > A (*p*.Gly186Glu) as previously described pathological mutations [20] and a so far unknown mutation on LPL Exon 5, c.659G > A (*p*.Ser220Asn). In one patient a compound heterozygous mutation was identified, carrying two different known genetic variants c.644G > A (*p*.(Gly215Glu)) and c.1019-3C > A [21].

## 4. Discussion

Familial chylomicronemia syndrome is characterized by severe fasting hypertriglyceridemia caused by dietary-derived chylomicrons [3]. FCS diagnosis in early childhood is important to avoid severe complications mainly related to pancreatitis. However, the treatment with strict fat reduction is highly challenging, especially in childhood and adolescents to guarantee growth and pubertal development and to minimize the risk of pancreatitis and related complications. Pharmacotherapy with fibrates, niacin and statins is usually not effective to decrease extremely elevated plasma triglycerides in FCS [3]. Therefore, specialized dietitians need to support the families in setting up a strict fat-reduced diet and the use of MCT-oil, correct supplement of essential fatty acids, fat-soluble vitamins and micronutrients as well as age-adapted food composition and meal preparation. Continuous balancing between the plasma lipids and fatty acid fractions is necessary.

Novel pharmacological therapies like inhibitors of APOC3 might be a future option also for children [22,23]. A lifelong strict diet is necessary for patients suffering from familial chylomicronemia syndrome. After diagnosis, immediate treatment by strict dietary fat reduction is required to lower the massively increased triglycerides. In severe cases, lipid apheresis is indicated and is used to prevent neurological or other abnormalities due to circulatory disorders [24,25]. In 25% of FCS patients, complications manifest already within the first year of life [3,26]. Moreover, triglyceride levels exceeding more than 2000 mg/dL are associated with a 10–20% risk to develop acute pancreatitis [27]. Our patients had excessively increased triglyceride levels at the baseline visit, in two patients triglyceride levels exceeded 30,000 mg/dL, one patient presented with approximately 10,000 mg/dL and one patient with about 2400 mg/dL. At diagnosis, all four patients suffered from several clinical symptoms. The greatest impact could be achieved immediately after diagnosis and under a very strict dietary regime in all patients. Strict fat limitation is necessary to keep the triglyceride levels in an acceptable range (about 1000 mg/dL) and to prevent clinical deterioration in childhood.

### 4.1. Recommended Implementation of FCS Nutrition

In 2018, Williams et al. published the guidelines for the daily fat intake for FCS patients and demanded a reduction of the fat fraction to 10–15% of the total caloric intake [6]. However, these recommendations are not feasible for the long-term therapy of pediatric patients and are not applicable for Austria, especially regarding the availability of products in the local supermarket. In addition to the low-fat diet, added sugars, sugar-sweetened beverages, fruits juices should be avoided according to the recommendation and eventually, there are major restrictions for this young vulnerable group of patients. Therefore, instead of two categories to “include” and to “avoid”, we have divided a detailed list of food and drinks by three categories in the traffic light system to have the option of allowed in moderate portion sizes. It has to be mentioned that the group of Williams et al. is from the United States of America. Thus, the current dietary recommendations, especially in terms of sample meals and dietary products are not feasible for European countries. The Americans recommend as sample meals fatty fish, chia seeds, powdered peanut butter and different spices and herbs. In our opinion, chia seeds contain too much fat, powdered butter is not available in local grocery stores in Austria and spices and herbs cannot be categorized as food. The issue of incompliance of children and adolescents is mentioned according to “picky eaters”. None of our patients could be defined as picky eaters or children with eating disorders. One child suffered from failure to thrive, but this was already observed from birth. From our experience, incompliance is not associated with “picky eaters” but is quite normal and can be observed in a lifelong diet that should be adhered to. The use of the lipase inhibitor orlistat is also recommended. However, this medicinal product is not approved for children in Austria.

The nutritional needs and eating habits of infants, toddlers, adolescents and adults differ fundamentally. A generalized recommendation cannot be used due to the differences in growth and development. Furthermore, nutrient and energy requirements as well as food portion sizes increase with the child’s age. We have managed to develop a dietary tool, the easily understandable traffic light system with age-appropriate portion sizes, which is easy to use for children as well as parents. In addition, individual needs should be considered, especially for the application and adherence of a lifelong diet. For example, we were able to reduce the fat intake of one patient to 3% of the total daily energy intake. However, this was only possible with a special fat-free formula food, which cannot be used as a long-term therapy. Another child suffered from failure to thrive from birth. Thus, dietetic therapy for this child included an increased dietary fat intake in order to guarantee adequate growth.

A described problem and also observed in our patients is the adherence of the lifelong very low-fat diet. Incompliance can result in an increase of plasma triglycerides rapidly and abdominal pain, as well as pancreatitis could occur as acute side effects. Patient 1 had repeated silent and non-reported pancreatitis episodes, which subsequently led to pancreatic atrophy with so far adequate exocrine and endocrine function. Retrospectively, it must be noted that the patient often failed to adhere to the diet and e.g., repeatedly consumed chocolate.

Risk situations to neglect the diet are celebrations and outside eating as well as demand for sweets and chocolate in childhood. Therefore, elevated triglyceride levels were observed in some control visits. Regular contact between the patient and the dietitian and retraining are most important to reduce diet failure. Hence, recommendations and implementation of a fat-reduced diet, especially in childhood with age specific nutritional requirements, must be conclusive, clear and easy. The current dietary recommendations for patients with FCS are based on limited observations and single expert opinions [6]. The recommendations contain fat-free milk and fat-free dairy products, which are not available in Austria. Therefore, our strategy covers a fat fraction reduction only to 10–26% of total daily energy intake, which seems to be feasible in long-term treatment in pediatric FCS patients. The amount of dietary fat intake of 20–25% was published as cut-off value of the total daily caloric intake [28].

During childhood, nutrition means an age-adapted food composition, in infancy breast milk and standard infant formulas, both containing a high amount of fat [6]. Hence, the use of special infant formulas with very low-fat and long-chain triglycerides content, but increased medium-chain triglycerides content, as well as sufficient essential fatty acids is recommended and has to be monitored [7]. Our patients received special dietary infant formula, Monogen (Nutricia, Austria) with a high-MCT ratio, or Milupa basic-F (Nutricia, Austria), which does not contain any medium-chain triglycerides and essential fatty acids were added. This is important to ensure adequate neurocognitive development [6]. For complementary feeding, dietary products (KeyOmega^®^ Vitaflo) or walnut/soy oil were added to meet the requirements for essential fatty acids. The supplementation of *n*-3 fatty acids was often used in FCS as it is safe and cost-efficient, but data on effectiveness are inconsistent [29,30,31]. Medium chain triglycerides are the preferred dietary fat source because they are directly absorbed to the portal vein and do not use chylomicron transportation [3]. MCT supplementation in small quantities is recommended to prevent abdominal pain, flatulence and diarrhea [7]. In our children with FCS, MCT supplementation was started with the complementary feeding period. That means bottle-feeding was replaced by a low-fat MCT-enriched porridge meal. Exact calculation of the fat amount is not necessary; therefore, the daily use of the traffic light table is easy. Food and drinks in the “green area” are recommended, but fat-rich food, sugars, nuts and alcohol must be avoided (“red area”). Food in the “yellow area” can be eaten in age-appropriate quantities. Especially for children there should be no additional restrictions such as exclusive consumption of wholemeal bread, fat-free sweets and juice.

One must consider that FCS is associated with a reduced quality of life and has a great impact on the patient and the family [32]. Ethnicity, religion and socio-economic status of the family play an important role in food choice and must be considered during nutritional counselling and for food selection. The fat-restricted diet in infancy and toddler’s age is controlled by the parents but is a great challenge in kindergarten and school. Training and information are necessary for the implementation of the strict diet and offer participation in everyday life.

### 4.2. Strengths and Limitations

The strength of this study is the long-term follow-up of four pediatric FCS patients from first diagnosis at infancy and their follow-up during childhood, and one up to adulthood. A traffic light table for pediatric FCS patients with support of age-appropriate portion sizes was developed as a tool for affected families. This study showed the differences between current dietary recommendations and the feasibility in children and adolescents with FCS, as well as the effects of the strategy used for pediatric FCS patients in our outpatient clinic.

A larger cohort of pediatric FCS patients and a long-term prospective trial might help to improve quality of life and therapeutic strategies for children and adolescents with FCS.

## 5. Conclusions

Current guidelines recommend a very low-fat diet of <10–15% of total daily fat intake in FCS patients. This recommendation, especially in long-term treatment, is not feasible in pediatric patients. In summary, a restriction of dietary fat fraction to 10–26% of the total daily caloric intake is feasible and effective in the long-term treatment of pediatric familial chylomicronemia syndrome patients. This dietary management can decrease triglycerides to approximately 1000 mg/dL, and strict adherence to the diet prevents FCS symptoms and the risk for acute pancreatitis is diminished. The dietary tool, the pediatric FCS traffic light table and the age-appropriate portion sizes for patients between 1 to 18 years, supports children and their parents to achieve and adhere to the lifelong strict low-fat diet.

## Figures and Tables

**Table 1:** Familial chylomicronemia syndrome traffic light table.

Food Selection for Pediatric Patients with Familial Chylomicronemia Syndrome		
**Green Traffic Light** You can eat and drink.	**Yellow Traffic Light** Pay attention to the amount. Age–appropriate portion size.	**Red Traffic Light** Do not eat and drink.
**Beverages**		
Tap water, mineral water, all types of tea, fruit juice mixed with water, diluted fruit syrup (also with maltodextrin or sugar in case of high energy requirement considering the glycemic index)	Fruit whey 0.1% fat Lemonade without added sugar (light products)	Alcoholic drinks, mixed milk drinks with a fat content over 1%
**Bread and Cereal Products**		
Bread without nuts and seeds, white bread, mixed bread, bread rolls, toast, wholemeal bread, cornflakes, pasta, rice, polenta without added fat, cereal flakes and mueslis without nuts and chocolate	Bread or rolls with seeds or nuts, yeast plaits	Croissant, chocolate croissant, chocolate donut, cheese straws, breakfast cereals with nuts, chocolate muesli
**Spreads**		
Spread cheese up to 0.2% fat, tomato paste	Home–made vegetarian spreads up to 3% fat, honey, jam	Chocolate hazelnut spread, peanut butter, humus, spreads over 3% fat
**Potato and Potato Products**		
Natural boiled potatoes, baked potatoes, salt potatoes, parsley potatoes without added fat, potatoes in the oven without fat, potato salad without oil	Mashed potatoes with milk (maximum 0.9% fat) and with medium chain triglyceride (MCT)–margarine/or MCT–oil	Fried potatoes, potato pancakes, french fries and croquettes prepared in the deep fryer, potato salad with oil or mayonnaise
**Spices**		
All (except those listed in the yellow and red–marked area)	Ketchup, mustard	Spicy sauces with added fat
**Vegetables and Salad**		
Fresh vegetables, frozen vegetables without added fat, all salads without added fat, canned vegetables without added fat, pure vegetable jars (complementary foods) without added fat (see list of ingredients) (maximum 0.5 g fat in 100 g). Beans (e.g. white, red), green peas, lentils (as tolerated)	Chickpeas	Vegetable sticks baked in fat, convenience products (e.g. roasted vegetables) with added cream or butter vegetables/creamed spinach, soy flour, soy beans tofu, olives
**Fruits and Nuts**		
Fresh fruit, frozen fruit, canned fruit, chestnuts, pure fruit glasses (complementary food)	Dried fruit: raisins, bananas, apple rings, etc. (note the list of ingredients)	All types of nuts such as almonds, walnuts, pistachios, hazelnuts, coconuts, etc. avocado
**Milk and Dairy Products**		
	Milk/yoghurt up to 1% fat, buttermilk, skimmed curd/curd cream up to 1% fat, sliced cheese: up to 25% fat in the dry matter = approx. 13 g absolute fat/100 g; cream cheese: up to 8% absolute fat, cottage cheese up to 1% fat, cocoa with milk up to 1% fat, homemade pudding with milk up to 1% fat	Yoghurt, milk, curd milk over 1% fat, curd cheese/curd cream over 1% fat, fruit yoghurt over 1% fat, cheese over 25% fat in the dry matter, pudding and cocoa over 1% fat, curd cheese, whipped cream, creme fraiche
**Meat, Sausage Products, Fish**		
	Turkey schnitzel, turkey breast, chicken breast (skinless) prepared without added fat, pork, veal, beef fillet or natural schnitzel prepared without added fat, beef (lean) minced meat, poultry sausages or sausages, ham cooked and raw without a fat rim, low–fat sausages up to 2% fat, low–fat fish e.g. saithe, cod, plaice, canned tuna in water, shrimp and prawns	Poultry with skin, breaded meat baked in fat, mixed minced meat, cheese filed sausages, Bernese sausage with bacon, Frankfurter sausages, pork sausage, ham, bacon, belly meat, liver sausage, salami, mortadella, blood sausage, meat loaf herring, salmon, mackerel, smoked fish, breaded fish baked in fat, canned tuna in oil, fish fingers baked in fat or in an oven
**Eggs and Egg Dishes**		
	maximum two egg yolks (eggs) per week (consider processed eggs in other food products)	Eggs fried in fat, scrambled eggs with bacon or sausage, ham and eggs
**Fats and Oils**		
	MCT–oil and walnut oil (soybean oil) – according to the diet plan, MCT–margarine according to the diet plan	Margarine, butter, mayonnaise, cooking oil
**Cakes, Sweets and Snacks**		
(Whole grain) rice waffles, chewing gum, homemade ice pops made of fruit juice	Low–fat yeast dough with fruit, sponge cake, yeast plaits, ladyfingers, fruit gums, candies, chewy candies, lollipops, marshmallows and foamy sugar, sorbet, pretzel sticks etc.	Pies with whipped cream, biscuits, butter biscuits, muesli bars, sponge cake, pastry, donuts, waffles, chocolate bars, chocolate, ice cream, milk ice cream, milk–based fruit ice cream, chips, peanut flips, crackers
**Convenience Food and Fast Food**		
Salads with low–fat dressing (max. 0.5% fat in 100 g) or marinated with MCT–oil, fruit puree and vegetable puree (“quetschies”) without added fat, smoothies without nuts, seeds and added fat	Instant soup and convenience food up to 1% fat	Hamburger, cheeseburger, Big Mac, McChicken, etc., gyros, doner kebab, crepes, pizzas with salami or bacon, chicken nuggets, hot dogs, ready–to–eat sauces with mayonnaise

**Table 2:** Age-appropriate portion sizes used for the yellow-marked area in the FCS traffic light table.

Age–Appropriate Portion Sizes	
Food Groups	Household Size or Child’s Hand to Determine 1 Portion size
Beverages	1 glass
Bread/Cereals	1 slices bread/2 hands full cereals
Vegetables	1–2 hands full
Fruits	1–2 hands full
Potato and Potato products	2 hands full
Milk and dairy products ^a^	
Milk	1 glass
Yoghurt	1 cup
Cheese	1 slice
Meat/Sausage products ^b^	1 palm of the child’s hand meat/1–3 slices of sausages
Eggs	1–2 eggs
Fish	1 palm of the child’s hand
Oil/Margarine/Butter/Spreads	1.5–2 table spoons
Spices	1–2 tea spoons
Cakes, Sweets/Snacks	1 piece cake, sweets/1 hand full snacks

^a^ 100 g milk or 100 g yoghurt correspond to the amount of 30 g cheese and can be converted. ^b^ The quantities given here assume that 2 servings of meat, 3 servings of sausage, 1 serving of fish and 1 serving of egg are eaten within a week.

**Table 3:** Patient 1—blood parameters and nutrients intake.

Patient 1, Female	Visit 1	Visit 2	Visit 3	Visit 4	Visit 5	Visit 6
**Patients Characteristics**						
Age (months)	1.6	2.0	58.2	69.0	99.3	169.0
Age (years)	0.1	0.2	4.8	5.8	8.3	14.2
Height (cm)	55.0	58.0	104.0	108.9	122.4	157.1
Weight (kg)	3.7	4.1	15.3	17.0	22.1	39.4
BMI (kg/m^2^)	12.2	12.0	14.0	14.2	14.7	16.0
**Blood Parameters**						
Triglycerides (mg/dL)	9859	290	1282	336	847	1866
Total cholesterol (mg/dL)	749	150	144	90	131	165
FCS clinical manifestations	Hypertriglyceridemia Pancreatitis Splenomegaly Cholecystitis	Hyper- triglyceridemia	Hyper- triglyceridemia	Hyper- triglyceridemia	Hyper- triglyceridemia	Hypertriglyceridemia Hepatomegaly Splenomegaly Gallbladder stone without cholecystitis Pancreas atrophy
Pharmacological therapy						Bezafibrate 400 mg Ursodeoxycholic acid 500 mg
**Nutrients** **Energy Percentage (%)**						
Fat (%)	47.7	14.6	27.2	26.0	25.8	32
Protein (%)	10.8	12.9	22.2	19.1	14.5	20.0
Carbohydrate (%)	41.5	72.3	49.0	53.2	57.6	46.6
Energy (kcal)	317	590	776	998	1366	1277
Energy (kcal/kg BW)	86	145	50	59	62	32
Fat (g)	16.8	9.6	23.6	28.9	39.2	45.5
Protein (g)	8.6	18.9	43.0	47.8	49.6	64.1
Carbohydrate (g)	32.9	106.7	93.9	132.8	196.8	148.9
MCT (g)	8.6	2.3	0.03	3.8	7.6	3.9
LCT (g)	7.6	7.3	23.4	25.0	31.5	41.6
n3-FA (g)	0.03	0.73	6.49	6.24	6.27	2.8
n6-FA (g)	2.3	1.5	3.6	5.3	5.1	6.8
PUFA (g)	2.9	2.2	10.1	11.5	11.4	9.6
Comments on nutrition	Prior to diagnosis: breastfeeding ad libitum than changed to formula feeding (481 g Aptamil Pregomin standard concentration 12.8%)	135 g fat-free formula (basicF powder, Milupa) 15%, 6 g essential FA 3 g MCT-oil (MCT basis plus)	6 g omega 3 FA capsules (Omacor) 8 g essential FA	Low-fat age- appropriated nutrition 24 g Maltodextrin 6 g omega 3 FA capsules (Omacor) 5 g MCT-oil (MCT basis plus oil) 6 g essential FA	Low-fat nutrition 30 g maltodextrin 10 g MCT-oil 6 g omega 3 FA capsules (Omacor) 5 g vitamin and micronutrients supplement (Metax Advit—corresponding to half of the daily vitamin and micronutrient requirement) 6 g essential FA	2 g omega 3 FA capsules (Omacor) 5 g MCT-oil 5 g vitamin and micronutrients supplement (Metax Advit—corresponding to half of the daily vitamin and micronutrient requirement)

BMI = body mass index; BW = body weight; FA = fatty acids; LCT = long chain triglycerides; MCT = medium chain triglycerides; PUFA = polyunsaturated fatty acids. Omacor = a drug containing eicosapentaenoic acid and docosahexaenoic acid.

**Table 4:** Patient 2—blood parameters and nutrients intake.

Patient 2, Male	Visit 1	Visit 2	Visit 3	Visit 4	Visit 5	Visit 6
**Patients Characteristics**						
Age (months)	1.0	1.0	1.0	2.0	6.8	72
Age (years)	0.08	0.1	0.1	0.2	0.6	6
Height (cm)	54.0	54.0	54.0	58.0	69.0	117.4
Weight (kg)	3.8	3.8	4.0	5.1	8.4	19.5
BMI (kg/m^2^)	13.0	13.0	13.7	15.1	17.5	14.1
**Blood Parameters**						
Triglycerides (mg/dL)	31,012	5001	1104	587	828	1668
Total cholesterol (mg/dL)	1402	1193		200	108	249
FCS clinical manifestations	Abdominal pain Bloody stool Hypertriglyceridemia Hepatomegaly Splenomegaly Steatosis hepatis Choleocystitis	Hyper- triglyceridemia Cholecystitis Steatosis hepatis	Hyper- triglyceridemia Steatosis hepatis	Hyper- triglyceridemia	Hyper- triglyceridemia	Hyper- triglyceridemia Hepatomegaly
Pharmacological therapy	Colecalciferol drops	Colecalciferol drops	Colecalciferol drops	Colecalciferol drops		Bezafibrate 100 mg
**Nutrients** **Energy Percentage (%)**						
Fat (%)	48.5	3.2	20	20.5	13.3	9.5
Protein (%)	6.9	14.5	11.6	11.6	12.4	21.7
Carbohydrate (%)	43.7	81.7	68.0	67.5	73.1	67.0
Energy (kcal)	414.0	309.0	423.3	539.2	827.8	1222
Energy (kcal/kg BW)	109	81	106	106	98	63
Fat (g)	22.3	1.1	9.4	12.3	12.1	12.9
Protein (g)	7.2	11.2	12.3	15.7	25.5	66.2
Carbohydrate (g)	45.2	63.1	72.0	91.0	151.2	204.6
MCT (g)	1.44	0.05	2.52	4.75	4.59	0.52
LCT (g)	20.9	1.05	6.88	7.56	7.62	12.4
n3-FA (g)	0.37	0.012	0.31	0.41	0.53	0.76
n6-FA (g)	0.12	0.004	1.09	1.61	2.37	3.80
PUFA (g)	2.8	0.1	2.1	2.6	3.5	4.6
Comments on nutrition	600 mL breastfeeding ad libitum	20 mL human milk and 78 g fat-free formula (basicF powder, Milupa)	100 mL human milk 78 g fat-free formula (basicF powder, Milupa) 5 mL MCT emulsion (Liquigen Nutricia) 4 g omega FA (Key Omega Vitaflo) 2 g essential FA	80 mL human milk 104 g fat-free formula (basicF powder, Milupa) 10 mL MCT emulsion (Liquigen Nutricia) 4 g omega FA (Key Omega Vitaflo) 3 g essential FA	473 g fat-free formula (basicF powder, Milupa, 13% standard concentration) 47 g Beba HA Start Pre 6 ml MCT emulsion (Liquigen Nutricia) 4 g omega FA (Key Omega Vitaflo) 3 g essential FA and age-appropriated complementary feeding	According to the recommendations no omega 3 substitution, strictly low-fat diet 4 g essential FA

BMI = body mass index; BW = body weight; FA = fatty acids; LCT = long chain triglycerides; MCT = medium chain triglycerides; PUFA = polyunsaturated fatty acids.

**Table 5:** Patient 3—blood parameters and nutrients intake.

Patient 3, Male	Visit 1	Visit 2	Visit 3	Visit 4	Visit 5	Visit 6
**Patients Characteristics**						
Age (months)	20.6	22.5	27.9	35.2	42.7	88.0
Age (years)	1.7	1.9	2.3	2.9	3.6	7.4
Height (cm)	82.0	83.0	86.0	91.0	92.6	114.7
Weight (kg)	8.3	8.8	9.4	10.0	10.9	16.4
BMI (kg/m^2^)	12.4	12.7	12.7	12.0	12.7	12.5
**Blood Parameters**						
Triglycerides (mg/dL)	2417	265	318	1796	1633	1358
Total cholesterol (mg/dL)	176	129	119	171	180	176
FCS clinical manifestations	Eruptive xanthomas Hypertriglyceridemia Hepatomegaly Splenomegaly Steatosis hepatis	Hypertriglyceridemia Steatosis hepatis	Hypertriglyceridemia Steatosis hepatis	Hypertriglyceridemia Steatosis hepatis	Hypertriglyceridemia Steatosis hepatis	Hypertriglyceridemia Steatosis hepatis Minimal increased IMT thickness in the area of arteria carotis communis on both sides
Pharmacological therapy					Colecalciferol drops	
**Nutrients** **Energy Percentage (%)**						
Fat (%)	32.8	10.4	24.7	24.7	23.7	22.4
Protein (%)	13.0	17.5	14.8	12.6	11.4	17.3
Carbohydrate (%)	52.4	70.2	58.4	51.5	62.7	58.5
Energy (kcal)	942	939	1073	1262	1114	1361
Energy (kcal/kg BW)	113	107	114	126	102	83
Fat (g)	34.3	10.8	29.5	34.6	29.3	33.9
Protein (g)	30.6	41.1	39.8	39.8	31.8	58.9
Carbohydrate (g)	123.3	164.6	156.6	194.0	174.6	198.9
MCT (g)	0.03	0.04	14.8	6.6	10.8	18.0
LCT (g)	34.3	10.8	14.7	28.0	18.5	15.9
n3-FA (g)	30.2	1.4	0.9	1.0	0.7	0.9
n6-FA (g)	7.9	2.8	3.7	5.3	4.6	4.0
PUFA (g)	11.5	4.2	10.6	12.3	5.3	4.8
Comments on nutrition	Prior to diagnosis 100 g formula (HA Combiotik) 3 g omega 3 FA capsules (Arteriomed) 6 g essential FA	45 g maltodextrin 1 g omega 3 fish oil 3 g essential FA	8 g MCT-oil (Nutricia) 1 g omega 3 fish oil 10 g MCT margarine 4 g essential FA	5 g MCT-oil (Nutricia) 1 g omega 3 fish oil 5 g essential FA	10 g MCT-oil (Nutricia) 2 g MCT powder (MCT Procal vitaflo) 2 g essential FA	15 g MCT-oil (Nutricia) 5 g essential FA

BMI = body mass index; BW = body weight; FA = fatty acids; IMT = Intima Media Thickness; LCT = long chain triglycerides; MCT = medium chain triglycerides; PUFA = polyunsaturated fatty acids.

**Table 6:** Patient 4—blood parameters and nutrients intake.

Patient 4, Female	Visit 1	Visit 2	Visit 3	Visit 4	Visit 5	Visit 6
**Patients Characteristics**						
Age (months)	2.0	2.5	56.3	147.6	162.0	218.0
Age (years)	0.2	0.2	4.7	12.3	13.7	18.0
Height (cm)	52.0	52.0	112.4	174.0	178.5	178.6
Weight (kg)	5.6	6.1	20.9	61.5	76.2	76.5
BMI (kg/m^2^)	20.6	22.5	16.5	20.3	23.9	24.0
**Blood Parameters**						
Triglycerides (mg/dL)	32,148	9540	1242	1138	953	1251
Total cholesterol (mg/dL)	1704	1130	169	197	131	239
FCS clinical manifestations	Bloody stool Lipemia retinalis Hypertriglyceridemia Hepatomegaly	Hypertriglyceridemia	Hypertriglyceridemia	Hypertriglyceridemia	Hypertriglyceridemia	Hypertriglyceridemia Splenomegaly
Pharmacological therapy				Bezafibrate 200 mg Colecalciferol drops	Bezafibrate 400 mg	
**Nutrients** **Energy Percentage (%)**						
Fat (%)	48.5	29.0	23.0	11.4	13.8	13.0
Protein (%)	7.0	10.1	22.2	27.9	13.0	15.2
Carbohydrate (%)	43.7	60.4	54.0	57.2	71.2	69.7
Energy (kcal)	414	629	1021	1833	1641	1399
Energy (kcal/kg BW)	74	103	49	29	22	18
Fat (g)	22.3	20.3	26.1	23.3	25.1	20.3
Protein (g)	7.2	15.8	56.7	127.7	53.2	53.1
Carbohydrate (g)	45.2	95.0	137.9	261.9	292.1	243.9
MCT (g)	1.44	10.84	0.01	0.12	0.90	0.6
LCT (g)	20.9	9.5	26.1	19.8	24.2	19.7
n3-FA (g)	0.37	0.12	1.42	4.24	0.66	1.91
n6-FA (g)	0.12	0.12	4.79	3.74	8.32	5.44
PUFA (g)	2.8	2.6	9.4	8.0	8.9	7.3
Comments on nutrition	600 mL breastfeeding ad libitum	100 g formula with primary MCT (Monogen Nutricia powder) 30 g standard formula (Milupa, powder) 10 g maltodextrin	1 g omega 3 FA capsules (Omacor)	4 g omega 3 FA capsules (Omacor)	Vegan diet	Vegan diet

BMI = body mass index; BW = body weight; FA = fatty acids; LCT = long chain triglycerides; MCT = medium chain triglycerides; PUFA = polyunsaturated fatty acids; Omacor = a drug containing eicosapentaenoic acid and docosahexaenoic acid.

## Data Availability

Data and intervention materials are available upon request to the corresponding author.
